# The contribution of prosody to machine classification of schizophrenia

**DOI:** 10.1038/s41537-024-00463-3

**Published:** 2024-05-18

**Authors:** Tomer Ben Moshe, Ido Ziv, Nachum Dershowitz, Kfir Bar

**Affiliations:** 1 Blavatnik School of Computer Science, Tel Aviv University, Tel Aviv, Israel; 2 Behavioral Sciences, Netanya Academic College, Netanya, Israel; 3 Effi Arazi School of Computer Science, Reichman University, Herzliya, Israel

**Keywords:** Biomarkers, Schizophrenia

## Abstract

We show how acoustic prosodic features, such as pitch and gaps, can be used computationally for detecting symptoms of schizophrenia from a single spoken response. We compare the individual contributions of acoustic and previously-employed text modalities to the algorithmic determination whether the speaker has schizophrenia. Our classification results clearly show that we can extract relevant acoustic features better than those textual ones. We find that, when combined with those acoustic features, textual features improve classification only slightly.

## Introduction

Schizophrenia is an acute mental disorder characterized by delusions, hallucina- tions, and thought disorders. Thought disorders are disturbances in the normal way of thinking, typically presented as various language impairments, such as disorganized speech, which are related to abnormal semantic associations between words^1^. These include the following: (1) poverty of speech; (2) pressure of speech, fast, loud and hard-to-follow responses; (3) “word salad”, random-word selection at times; (4) derailment, shifting from one topic to another during a conversation; and (5) tangentiality, furnishing an irrelevant response, never reaching the answer to the posed question^2^. Andreasen^3^ provides some statistics for symptoms of thought disorder, with the most common being derailment, loss of goal, poverty of content, and tangentiality.

Diagnosing thought disorders is performed by clinicians and mental-health professionals, typically by means of a conversation. This is an arduous and subjective process. Mental-health professionals are on constant lookout for objective computational assessment tools that can help identify whether a person is showing signs of thought disorders.

There have been several prior attempts at developing computational tools for analyzing language with the goal of detecting symptoms of mental-health disorders; we describe some of those works in the following section. Generally speaking, speech and text are the two modalities of human language that can be processed and analyzed algorithmically for the diagnosis of mental-health disorders. For this purpose, processing speech is typically done for the purpose of modeling the prosody by extracting features related to intonation, stress and rhythm. One of the most prominent prosodic symptoms is flattened intonation, or aprosody, which is interpreted as inability of a person to properly convey emotions through speech. This is a negative symptom of schizophrenia. Another negative symptom that is associated with speech is alogia, or poverty of speech, presented as very minimal speech. Metaphorically, it has been claimed^4,5^ that patients with schizophrenia sometimes sound like a person talking on the phone, referring to the low-quality aspect of the voice, sometimes occasionally to as a “creaky” voice. Cohen et al.^6^ associate acoustic-based analysis of speech, generally speaking, with clinically rated negative symptoms, while associations with positive symptoms have been found to be inconsistent.

Prosody, which encompasses aspects of language beyond the scope of grammar and vocabulary choice, can reflect subtle elements such as emotions and pragmatic nuances. Conversely, the transcription is essential for capturing the linguistic and semantic characteristics inherent in conversations. It’s important to note, however, that non-emotional aspects of prosody also exist and play a significant role in communication.

We study the salience of acoustic and textual features for the classification task of automatically detecting whether a given utterance was generated by someone who has been diagnosed with schizophrenia or by a control subject. To do that, we measure the contribution of each set of features once when used individually for classification, and again when both modalities are combined together.

Our dataset comprises transcribed interviews, collected from native Hebrew-speaking inpatients, officially diagnosed with schizophrenia at a mental health center in Israel, and from a demographically balanced control group. The prosodic features that we consider are based on pitch, which we extract using an audio processor. The textual features are extracted from the transcriptions of the audio files and are designed to capture symptoms such as derailment and incoherence, following a previous work^7^ that has shown the efficacy of such features when used in a similar classification task.

Prosodic features have been computationally examined previously and were shown to be effective for the task of detecting schizophrenia—for example^8,9^ for English speech. For Chinese, Huang et al.^10^ combined acoustic features with textual features for assessing the severity of thought disorders in examined schizophrenia patients. However, none of these works compare the individual contributions to classification of each of the modalities when used in combination.

Our contribution is twofold: (1) We show how acoustic prosodic features can be used for detecting symptoms of schizophrenia from only a single spoken response (given in Hebrew); and (2) we measure the individual contribution of both speech and text modalities to the task of detecting whether the person who generated a given utterance has schizophrenia. Our classification results clearly show that the acoustic prosodic features capture more information than do the textual ones. When combined with those acoustic features, textual features improve classification very slightly.

## Related work

The extensive literature about language characteristics and schizophrenia is examined in^11^. The authors distinguish between two types of language impairment among patients with schizophrenia: thought disorder—defined as disturbances in the normal way of thinking, and schizophasia—comprising various dysphasia-like impairments such as clanging, neologism, and unintelligible speech. They also assert that patients with thought disorders produce and perceive sounds in an abnormal way, manifesting as flat intonation or unusual voice quality.

Hoekert et al.^12^ conducted a meta-analysis of seventeen studies between 1980 and 2007. They found that prosodic expression of emotions is significantly impaired with schizophrenia. Martínez-Sánchez et al.^13^ compared the speech of 45 medicated schizophrenia patients and 35 healthy controls, all native Spanish speakers from Spain. The results revealed that patients paused more, talked more slowly, and showed less variability in speech and fewer variations in syllable timing. Alpert et al.^14^ examined whether “flat affect”, defined as emotionless speech, which is one of the symptoms of schizophrenia, indicates an emotional deficiency or whether this is only a communication issue. They did not find evidence for impairment in any other aspect of emotion expression besides prosody.

There is a large body of work that studies the efficacy of computational approaches for diagnosis of mental-health disorders. We continue by listing some related work that use computational tools to process acoustic speech signals for diagnosis of mental-health disorders, followed by works that use natural-language processing tools for analyzing transcriptions for the same purpose.

In a systematic review^15^ that analyzes 127 studies, the authors conclude that speech processing technologies could aid mental-health assessment; however, they mention several caveats that need to be addressed, especially the need for comprehensive transdiagnostic and longitudinal studies. Given the diverse types of datasets, feature extraction procedures, computational methodologies, and evaluation criteria, they provide guidelines for both data acquisition and building machine-learning models for diagnosis of mental-health disorders.

Kliper et al.^8^ trained a support vector machine (SVM) classifier that gained about 76% accuracy in a binary classification task of identifying people with schizophrenia versus controls, using acoustic features. The study population comprized 62 English-speaking participants, divided into three groups: patients with schizophrenia, patients with clinical depression, and healthy controls. In a three-way classification task over the three groups, their classifier achieved about 69% accuracy. Every participant was interviewed and recorded by a mental-health professional. Each recording was divided into segments of two minutes each, which were subsequently analyzed independently. Each recording was represented by nine acoustic features based on pitch and power, which were automatically extracted using tools similar to those that we use in this work.

Dickey et al.^16^ study prosodic abnormalities in patients with schizoid personality disorder (SPD). Their experimental results showed that SPD patients speak more slowly, with more frequent pauses, and exhibited less pitch variability than control participants.

A new algorithm to detect schizophrenia was proposed by^17^ based on a classifier that uses three new acoustic prosodic features. On a dataset comprised of 28 schizophrenia patients and 28 healthy controls, they measured classification accuracy between 89.3% and 94.6%.

Agurto et al.^18^ predict psychosis in youth using various acoustic prosodic features, such as pitch-related and Mel-frequency cepstral coefficients (MFCC). They analyzed the recorded speech of 34 young patients who were diagnosed to be at high risk of developing clinical psychosis. Among other things that they showed, they trained a classifier that can predict the development of psychosis with 90% accuracy, outperforming classification using clinical variables only.

Lucarini et al.^19^ offer a review of research papers focusing on the less-explored topic of non-emotional prosody. They introduce a linguistic model designed to classify prosodic functions along a continuum ranging from “linguistic,” pertaining to the structural aspects of language, to “paralinguistic,” which relates to the expression of emotions.

Lucarini et al.^20^ conducted an analysis of conversations between patients with schizophrenia and interviewers, aiming to detect associations between symptoms of schizophrenia and conversation dynamics. The approach centered on a relatively straightforward representation of a conversation, primarily encoding pauses and participant involvement. Their findings indicate a significant association between the dynamics of these conversations and negative symptoms of schizophrenia.

There has been an increasing number of works that computationally process speech transcriptions for detecting symptoms of schizophrenia. Specifically, measuring derailment and tangentiality has been addressed several times. For example, Elvevåg et al.^21^ analyzed transcribed interviews of inpatients with schizophrenia by calculating the semantic similarity between the response given the participants and the question that was asked by the interviewer. For semantic similarity they used cosine similarity over the latent semantic analysis (LSA) vectors^22^ calculated for each word, and summed across a sequence of words. Similarly, Bedi et al.^23^ use cosine similarity between pairs of consecutive sentences, each represented by the element-wise average vector of the individual words’ LSA vectors, to measure coherence. Using this score they automatically predicted transition to psychosis with perfect accuracy. Iter et al.^24^ showed that removing some functional words from the transcriptions improves the efficiency of using cosine similarity over LSA vectors for measuring derailment and incoherence.

This direction was developed further by Bar et al.^7^, who used fastText vectors^25^ to measure derailment in a study group that included 24 schizophrenia patients and 27 healthy controls, all native Hebrew speakers. Furthermore, they developed a new metric for measuring some aspects of incoherence, which compares the adjectives and adverbs that are used by patients to describe some nouns and verbs, respectively, with the ones used by the control group. As a final step, they used derailment and incoherence scores as features for training a classifier to separate the two study subgroups. In another work^26^ on the same study group, the authors used part-of-speech tags, lemma-to-token ratio, and some other morphological features, to perform a two-way classification for patients and controls. They report almost 90% accuracy.

We study a similar group of Hebrew-speaking male schizophrenia patients and healthy controls. Therefore, we use some of the same textual features suggested in that prior work to measure their respective contributions when combined with acoustic features.

Corona-Hernández et al.^27^ analyzed speech transcriptions of Dutch-speaking schizophrenia patients and controls, focusing on how connectives serve as informative and explainable variables. That study aimed to determine the reliability of using connectives to assess disorganized speech in patients with schizophrenia.

Finally, Corcoran et al.^28^ present a survey of various studies that employ similar techniques for measuring symptoms of psychosis and schizophrenia, by automatically analyzing speech transcriptions.

## Methodology

### Participants and data collection

We interviewed 48 men, aged 18–60, divided into control and patient groups, all speaking Hebrew as their first language. The patient group includes 23 inpatients from the Be’er Ya’akov–Ness Ziona Mental Health Center in Israel who were admitted following a diagnosis of schizophrenia. Diagnoses were made by a hospital psychiatrist according to the DSM-5 criteria (American Psychiatric Association, 2013) and a full psychiatric interview. Each participant was rewarded with approximately $8. The control group includes 25 men, mainly recruited via an advertisement that we placed on social media. The demographic characteristics of the two groups are given in Table 1. Exclusion criteria for all participants were as follows: (1) participants whose mother tongue is not Hebrew; (2) having a history of dependence on drugs or alcohol over the past year; (3) having a past or present neurological illness; and (4) using fewer than 500 words in total in their transcribed interview. Additionally, the control group had to score below the threshold for subclinical diagnosis of depression and post-traumatic stress disorder (PTSD). Most of the control participants scored below the threshold for anxiety. Most of the patients scored above the threshold for borderline or mild psychosis symptoms on a standard measure. (Our patient group is composed of inpatients who are being treated with medications; therefore, higher scores were not expected.) See Section 3.2 for more details about the assessment measures used in this study.

**Table 1:** Demographic characteristics by group.

	Control	Patients	Statistics
Subjects (N)	25	23	
Age mean (SD)	33.15 (9.98)	25.46 (6.39)	*t* = 3.24**
Years of education mean (SD)	11.96 (0.20)	11.21 (1.12)	*t* = 3.41**
Place of residence (frequencies)			*x*^2^(3, 49) = 8.29*
Southern Israel	1	7	
Central Israel	21	16	
Northern Israel	2	0	
Jerusalem	1	0	
Marital status (frequencies)			*x*^2^(1, 47) = 0.08, *p* = 0.77
Single	4	3	
Married	21	20	
PANSS positive subscale		9.21 ± 3.70	
PANSS negative subscale		8.26 ± 3.36	
PANSS total subscale		17.47 ± 5.52	

*p < 0.05; **p < 0.005.

The patients were interviewed in a quiet room at the department where they are hospitalized by one of our professional team members, and the control participants were interviewed in a similar room outside the hospital. Each interview lasted approximately 60 min. The interviews were recorded and later manually transcribed by a native Hebrew speaking student from our lab. All participants were assured of anonymity, and told that they are free to end the interview at any time.

After signing a written consent, each participant was asked to describe 14 black and white images picked from the Thematic Appreciation Test (TAT) collection. We used the TAT images identified with the following serial numbers: 1, 2, 3BM, 4, 5, 6BM, 7GF, 8BM, 9BM, 12 M, 13MF, 13B, 14, and 3GF. These include a mixture of men and women, children, and adults. The images were presented one by one. Each picture stands by itself, was presented alone, and bears no relation to the other pictures. Participants were asked to tell a brief story about each image based on four open questions:

(i) What led up to the event shown in the picture?
(ii) What is happening in the picture at this moment?
(iii) What are the characters thinking and feeling?
(iv) What is the outcome of the story?
  The interviewer remained silent during the respondent’s narration and offered no prompts or additional questions.
  After describing the images, the participant was also asked to answer four open-ended questions, one by one:
  1. Please tell me as much as you can about your *bar mitzvah*. (The Jewish confirmation ceremony for boys upon reaching the age of 13)
  2. What do you like to do, mostly?
  3. What are the things that annoy you the most?
  4. What would you like to do in the future?

As before, the interviewer remained silent during the respondent’s narration and offered no prompts or questions.

Once all 18 components (14 image descriptions and 4 open questions) were answered, each participant was requested to fill in a demographic questionnaire as well as some additional questionnaires for assessing mental-health symptoms, which we describe in the following subsection.

*NB. This research was approved by the Helsinki Ethical Review Board (IRB) of the Be’er Ya’akov–Ness Ziona Mental Health Center*.

### Symptom assessment measures

#### Control group

The control participants were assessed for symptoms of depression, PTSD, and anxiety.

##### Depression

Symptoms of depression were assessed using Beck’s Depression Inventory-II (BDI-II)^29^. The BDI-II is a 21-item inventory rated on a 4-point Likert-type scale (0 = “not at all” to 3 = “extremely”), with summary scores ranging between 0 and 63. Beck et al.^29^ suggest a preliminary cutoff value of 14 as an indicator for mild depression, as well as a threshold of 19 as an indicator for moderate depression. BDI-II has been found to demonstrate high reliability^30^. We used a Hebrew version^31^.

##### PTSD

Symptoms of PTSD were assessed using the PTSD checklist of the DSM-5 (PCL-5)^32^. The questionnaire contains twenty items that can be divided into four subscales, corresponding to the clusters B–E in DSM-5: intrusion (five items), avoidance (two items), negative alterations in cognition and mood (seven items), and alterations in arousal and reactivity (six items). The items are rated on a 5-point Likert-type scale (0 = “not at all” to 4 = “extremely”). The total score ranges between 0 and 80, provided along with a preliminary cutoff score of 38 as an indicator for PTSD. PCL-5 has been found to demonstrate high reliability^33^. We used a Hebrew translation of PCL-5^34^.

##### Anxiety

Symptoms of anxiety were assessed through the State Trait Anxiety Inventory (STAI)^35^. The STAI questionnaire consists of two sets of twenty self-reporting measures. The STAI measure of state anxiety (S-anxiety) assesses how respondents feel “right now, at this moment” (e.g., “I feel at ease”; “I feel upset”), and the STAI measure of trait anxiety (T-anxiety) targets how respondents “generally feel” (e.g., “I am a steady person”; “I lack self-confidence”). For each item, respondents are asked to rate themselves on a 4-point Likert scale, ranging from 1 = “not at all” to 4 = “very much so” for S-anxiety, and from 1 = “almost never” to 4 = “almost always” for T-anxiety. Total scores range from 20 to 80, with a preliminary cutoff score of 40 recommended as indicating clinically significant symptoms for the T-anxiety scale^36^. STAI has been found to have high reliability^37^. We used a Hebrew translation^38^.

#### Patients

Psychosis symptoms were assessed by the 6-item Positive And Negative Syndrome Scale (PANSS-6)^39^. The original 30-item PANSS (PANSS-30) is the most widely used rating scale in schizophrenia, but it is relatively long for use in clinical settings. The items in PANSS-6 are rated on a 7-point scale (0 = “not at all” to 6 = “extremely”). The total score ranges from 0 to 36, with a score of 14 representing the threshold for mild schizophrenia, and a score between 10 and 14 defined as borderline disease or as remission. PANSS-30 has been found to demonstrate high reliability^40^, while Østergaard et al.^39^ reported a high correlation between PANSS-6 and PANSS-30 (Spearman correlation coefficient = 0.86). We used the Hebrew version of PANSS-6 produced by Katz et al.^41^. The range of positive and negative symptoms are presented in the last three rows of Table 1.

### Data analysis

We analyse the data using two modalities, audio and text. All the interviews were recorded with a voice recorder, which was placed on the table next to the participant. The responses of the participants for each of the 18 interview components were recorded separately, and stored as individual files in Waveform Audio File Format (WAV). Each response was manually transcribed. We extracted prosodic acoustic features from the audio signal, as well as textual features from the corresponding transcriptions.

#### Prosodic acoustic features

We processed each WAV file with PRAAT^42^, a computer software package for speech analysis, in order to extract pitch and intensity per 10 ms frame. We use the PRAAT “Sound: To Pitch” method, assigned with its standard values, to detect frames with fundamental-frequency (F0) above 75 Hz. Typically, males’ pitch ranges between 75 Hz and 180 Hz and females’ from 80 Hz to 250 Hz. Furthermore, we noticed that some external noises occur in high frequencies. Therefore, we distinguish between speech and non-speech frames by automatically annotating as speech those frames with a detected F0 value above 75 Hz and below 250 Hz. Overall, we processed 18,187,506 10 ms frames, corresponding to approximately 50 h of recordings, out of which 8,377,628 frames had an F0 above 75 Hz. Only 322,189 (approximately 4% of 8 M frames) were above the 250 Hz threshold, resulting in 8,055,439 frames that we treated as carrying human speech. We acknowledge that errors may have occurred during the pitch-extraction process; we did not employ any correction utilities for the extraction. Additionally, we are aware that the voiceless sounds characteristic of Hebrew could potentially lead to some frames being misclassified as non-speech.

Each WAV file, corresponding to a response to a single image/question, is now represented by a sequence of speech frames, each represented by a pair of pitch and intensity values. We extract nine feature types from each response; to avoid overfitting, we filter out responses representing less than 10 seconds worth of speech. Therefore, we work with a dataset containing 449 responses given by controls and 409 responses given by patients. Following previous work on computational prosodic analysis^8^, we extracted the following set of features:

##### Mean Utterance Duration (MUD)

Every segment of at least 500 ms of continuous speech is defined as an *utterance*. MUD is the mean duration (in ms) of all the utterances in a given response. The threshold of 500 ms corresponds to 50 consecutive frames with a pitch value indicative of speech. Considering our criteria for identifying speech within a frame, there is a potential for omission of speech signals that could have been analyzed. However, given our focus—in this study—on comparing textual and acoustic features, we chose to concentrate on speech segments with a high likelihood of containing substantive content for meaningful extraction of both feature types.

##### Mean Gap Duration (MGD)

A *gap* is defined as a maximal time interval containing no speech. MGD is the mean length (in ms) of all gaps in a given response.

##### Mean Spoken Ratio (MSR)

The sum of the durations of all utterances in a response divided by the total response duration.

##### Mean Spoken Ratio Samples (MSRS)

The number of frames that are classified as speech divided by the total number of frames in the response.

##### Mean Pitch (MP)

The mean pitch (in Hz) of all frames recognized as speech in a given response.

##### Pitch Range (PR)

The maximum pitch of all frames recognized as speech, minus their minimum value, and divided by MP for normalization.

##### Standard Deviation of Pitch in a Single Response (PS)

The standard deviation of pitch (in Hz) of all frames recognized as speech in a given response.

##### Frame Pitch Correlation (FPC)

The Pearson correlation between a sequence of pitches of speech frames and a sequence of pitches of their consecutive frames in a given response. FPC, the way it is applied on pitch, measures the level at which the speaker sustains constant pitch. FPC is equivalent to mean waveform correlation (MWC), suggested in ref. ^8^.

##### Jitter (J)

The local deviation from stationarity of pitch. Formally, let *R* be the number of speech frames, and let *p*(*v*) be the pitch of the *v*th frame. We define J as follows:

$${\rm{J}}{\rm{:= }}\frac{1}{R-K}\mathop{\sum }\limits_{v=\frac{K-1}{2}}^{R-\frac{K-1}{2}-1}\frac{p\left(v\right)-\frac{1}{K}{\sum }_{k=-\frac{K-1}{2}}^{\frac{K-1}{2}}\,p(v+k)}{{\sum }_{k=-\frac{K-1}{2}}^{\frac{K-1}{2}}\,p(v+k)}$$

*K* is a locality parameter; it was set to 5 in all our experiments. Jitter quantifies the variability of a given measurement within a specific local context, determined by the locality parameter *K*. In other words, it assesses the stability of the time period within an environment spanning five consecutive frames.

We did not extract features that are based on intensity since we noticed some differences in the background noise between the recordings of the control participants and the patients, probably due to differences in room settings and recording equipment.

We verified that all the features are distributed normally, as expected, and performed *t* tests to measure the difference in feature expression between patients and controls. The results are summarized in Table 2. As can be seen, all the features associated with speech rate (MUD, MGD, MSR, MSRS) are distributed significantly differently among patients and controls. MGD exhibits relatively high levels of variability as indicated by the relatively large standard deviation. Consistent with other research^8^, our findings indicate that controls generally exhibit more fluent speech, characterized by significantly shorter pauses. These results should be qualified by a reminder that we consider only utterances that comprise at least 50 consecutive pitch frames marked as speech. Therefore, an MSR value of 0.124 (12%) for the patients does not necessarily mean that the patients speak for only 12% of the response time on average. It primarily means that only 12% of the signal is treated as substantive speech. Consequently, there is no intention to use the findings in Table 2 to draw direct conclusions about the speech patterns of participants. We primarily use these values as features for classification, as explained below. Conversely, analysis reveals that the mean pitch (MP) and pitch standard deviation (PS) of a given response are relatively comparable across the two groups. Nevertheless, more nuanced metrics derived from pitch values, such as jitter (J), frame pitch correlation (FPC), and pitch range (PR), demonstrate a more pronounced distinction between the groups. Upon examination of the jitter calculation methodology, it becomes apparent that the differences in pitch across consecutive frames may significantly contribute to the differentiation between the two groups. The explanation is the fact that jitter is calculated locally over five consecutive frames as defined by the locality parameter. The standard deviation of PS (5.517 and 7.370 for control and patients, respectively) indicates that there are some responses with high pitch variability, which in turn facilitates greater jitter variability. We ascribe the same explanation to the pronounced difference in FPC between the two groups, resulting in a more significant difference between them. The FPC values (0.581 and 0.483 for controls and patients, respectively) suggest that control participants exhibit a more consistent prosody while responding to questions compared to patients.

**Table 2:** Mean (SD) values of all prosodic features.

Feature	Control mean (SD)	Patient mean (SD)	*t* test	*p*
MUD	0.558 (0.120)	0.470 (0.151)	9.488	*<*0.001***
MGD	0.236 (0.083)	0.928 (1.502)	−9.739	*<*0.001***
MSR	0.446 (0.135)	0.220 (0.154)	22.831	*<*0.001***
MSRS	0.563 (0.100)	0.311 (0.138)	7.254	*<*0.001***
MP	129.202 (22.113)	125.422 (21.274)	0.602	0.550
PR	1.148 (0.153)	0.906 (0.196)	4.809	*<*0.001***
PS	21.579 (5.517)	19.411 (7.370)	1.160	0.252
FPC	0.581 (0.096)	0.483 (0.147)	2.750	0.009*
J	0.008 (0.002)	0.007 (0.002)	2.066	0.044*

*p < 0.05; ***p < 0.001.

We also experimented with a different method of calculating pitch range, dividing by the minimum pitch value instead of the mean value. The average and standard deviation for the control participants and patients using this alternate calculation were 1.814 (0.226) and 1.367 (0.383), respectively. This also resulted in a significant difference [*t* = −4.96 (*p* < 0.001***)] between the two groups. However, the distribution of this new pitch range variation did not differ markedly from the original.

#### Textual features

We extract the same textual features that have been used by Bar et al.^7^ on a similar dataset. Essentially, they designed two types of features for capturing specific symptoms of thought disorder.

##### Derailment

The first type is designed to capture derailment, which is a symptom of thought disorder when the speaker digresses from the main topic. Technically speaking, we represent words using static embeddings provided by fastText^43^ for Hebrew. For each response, we retrieve the fastText vector *v*_*i*_ for every word *R*_*i*_, *i* = 0*..n*, in the response. Then, for each word, we calculate a score defined as the average pairwise cosine similarity between this word and the *k* following words, with *k* a variable parameter. The score of a response is the average of all the individual cosine-similarity scores. To filter out functional words that do not contribute to the topical mutation assessment, we follow^7^ by pre-processing each response with a Hebrew part-of-speech tagger^44^ and keep only content words, which we take to be nouns, verbs, adjectives, and adverbs.

We calculate derailments for *k* = 1..6, thereby extracting six derailment features per response.

##### Incoherence

One of the most informative features reported in^7^ was designed to capture some aspects of discourse related to incoherence. Specifically, this feature examines the way patients use adjectives to describe specific nouns. The goal is to measure the difference between adjectives used by patients and the ones used by controls when describing the same nouns. Technically speaking, we process each response with YAP^45^, a dependency parser for Modern Hebrew, to find all noun-adjective pairs (indicated by the *amod* relation). To measure the difference between adjectives that are used by patients and controls, we compare them to the adjectives that are commonly used to describe the same nouns. To do that, following the above-mentioned work, we use an external corpus of health-related documents and forums, all written in Hebrew, containing nearly 680K words. (We use the same sources as in^7^). We process each document in exactly the same way to find all noun- adjective pairs. Given a list of noun-adjective pairs from one response, we calculate the similarity score between every adjective that describes a specific noun and the set of adjectives describing exactly the same noun across the entire external corpus. Hebrew enjoys a rich morphology; therefore, we work on the lemma (base-form) level. The lemmata are provided by YAP. We take the fastText vectors of the adjectives that were extracted from the external corpus and average them, element wise, into a single vector by assigning weights to each individual vector.

The weights are the inverse-document-frequency (idf) score of each adjective, to account more heavily for adjectives that describe the noun more uniquely. Then, we take the cosine similarity between each adjective from the response and the aggregated vector of the adjectives from the external corpus. For each response, we take the average of the individual adjective cosine-similarity scores as the overall response incoherence score.

As before, we verified that all our features are distributed normally and performed *t*-tests to measure the difference in feature expression between patients and controls. The results are summarized in Table 3. In contrast with the outcomes in^7^, we see no evidence for different distributions of each individual textual feature between the two groups. The datasets, patients and controls, differ for the two experiments. In^7^, the controls were told to talk for at least two minutes, which potentially impacted the outcome.

**Table 3:** Mean (SD) values of the textual features.

Feature	Control mean (SD)	Patient mean (SD)	*t* test	*p*
Derailment 1	0.247 (0.011)	0.239 (0.017)	1.797	0.080
Derailment 2	0.237 (0.015)	0.236 (0.013)	0.102	0.918
Derailment 3	0.233 (0.015)	0.231 (0.017)	0.297	0.768
Derailment 4	0.229 (0.015)	0.226 (0.021)	0.522	0.605
Derailment 5	0.227 (0.016)	0.226 (0.016)	0.331	0.742
Derailment 6	0.225 (0.016)	0.225 (0.016)	0.006	0.995
Incoherence	0.520 (0.062)	0.502 (0.070)	0.931	0.357

### Classification

We train a two-way machine-learning classifier to distinguish between responses that were generated by patients and controls. Each response is used as a classification instance, assigned either a “patient” or “control” label depending on the group to which the subject who generated the response belongs. Overall we have 449 responses generated by controls and 409 responses by patients. We ran three sets of experiments: (1) using only the acoustic features (Acoustic); (2) using only the textual features (Textual); and, (3) using both feature sets (Combined). Consequently, each response is represented by a nine-dimensional vector in the first set of experiments, a seven-dimensional vector in the second set, and a 16-dimensional vector in the third set of experiments.

For classification, we used three traditional machine-learning algorithms: XG-Boost^46^, Random Forest^47^, and Linear SVM^48^.

## Results

We measured the classification results using accuracy and the F1 score of the patient label. For each classifier, we ran five evaluations, each time taking a five-fold cross-validation approach. Every evaluation had a different random seed, which was kept similar across all classifiers. The five results were calculated as the average over the five evaluation runs. The results, divided into the three feature sets, are presented in Table 4.

**Table 4:** Classification results.

Feature Set	Classifier	Accuracy (SD)	Precision (SD)	Recall (SD)	F1 (SD)
Acoustic	Random Forest	88.4 (2.0)	87.3 (4.6)	80.3 (4.2)	80.8 (4.0)
Acoustic	XGBoost	90.1 (1.4)	95.3 (2.3)	86.0 (2.7)	87.8 (1.3)
Acoustic	Linear SVM	83.5 (2.0)	86.6 (4.0)	79.6 (4.7)	80.1 (3.2)
Textual	Random Forest	63.3 (1.5)	60.8 (1.4)	49.0 (5.1)	51.1 (7.4)
Textual	XGBoost	63.1 (4.8)	64.3 (5.7)	60.6 (4.8)	59.5 (5.4)
Textual	Linear SVM	73.4 (2.7)	74.0 (5.0)	65.3 (4.9)	66.4 (3.6)
Combined	Random Forest	89.0 (1.5)	91.6 (3.3)	83.6 (4.3)	85.3 (2.9)
Combined	XGBoost	88.5 (1.8)	94.0 (3.8)	81.3 (3.8)	84.8 (3.7)
Combined	Linear SVM	84.7 (1.8)	88.3 (4.0)	76.0 (4.6)	79.1 (3.6)

Overall, the XGBoost algorithm achieves the best classification performance when utilizing solely the acoustic features. When using only the textual features, all the classifiers perform poorly. Furthermore, combining the textual features with the acoustic ones did not usually result in significant performance improvement, suggesting that the contribution of our textual features to the classification performance on the dataset is limited and redundant when the acoustic features are used for detecting symptoms of schizophrenia. The lesser success with textual features may be due in part to the inherent difficulty of accurately measuring semantic features like derailment and incoherence computationally.

Our best accuracy for the two-way classification task is around 90%, which is higher than the best accuracy of about 76% reported by Kliper et al.^8^ using a similar set of acoustic features for the same two-way classification task with an English-speaking population.

Looking at the demographic characteristics of the participants in Table 1, we notice that the patients and controls significantly differ in age and years of education. Therefore, we performed a complementary analysis to support the current findings in which seven sets of multiple regressions have been carried out as reported in Table 5. These represent the seven prosodic features which demonstrated a significant (at least *p* < 0.05) different distribution among patients and controls. As shown in Table 3, none of the textual features has been shown to be different among the two groups.

**Table 5:** Seven regression analyses for the most impacting acoustic features.

	MUD		MGD		MSRS		MSR		PR		FPC		J	
	*V*	*t*	*V*	*t*	*V*	*t*	*V*	*t*	*V*	*t*	*V*	*t*	*V*	*t*
Age	−0.04	0.34	−0.29	2.04*	0.05	0.49	−0.08	0.65	0.24	1.88	0.16	1.04	0.18	1.1
Edu.	0.06	0.46	−0.05	0.38	0.07	0.62	0.13	1.02	−0.17	1.30	−0.10	0.68	−0.06	0.42
Grp.	0.47	3.04**	0.57	3.76***	0.72	5.87***	0.53	3.85***	0.76	5.4***	0.49	2.98**	0.4	2.35*
PEV	*R*^2^ = 0.28		*R*^2^ = 0.29		*R*^2^ = 0.54		*R*^2^ = 0.41		*R*^2^ = 0.41		*R*^2^ = 0.17		*R*^2^ = 0.11	
	*F* (3, 45) = 5.63**		*F* (3, 45) = 5.95**		*F* (3, 45) = 17.20***		*F* (3, 45) = 10.4***		*F* (3, 45) = 10.19***		*F* (3, 45) = 3.05*		*F* (3, 45) = 1.93	

*PEV* percentage of explained variance, *Edu.* Years of education, *Grp.* Group (patients/control). For more information, see the text. *p < 0.05,

**p < 0.01, ***p < 0.001.

As can be seen from Table 5, years of education consistently did not associate with any of the prosodic features. The age characteristic was associated significantly (*p* = 0.047) only once with MGD. However, the group (patients/control) was the only predictor that was associated consistently, substantially, and significantly with all the prosodic features.

We acknowledge potential variability in patient interactions during the interviews, as recently demonstrated in a study by Cangemi et al.^49^, which analyzed both speech and non-speech segments of patients with schizophrenia. Additionally, while the average PANSS-total score in our study is 17.34, above the threshold of 14, the considerable standard deviation of 6.29 indicates the presence of borderline cases and possibly some patients in remission. Specifically, among our sample of 23 patients, two have PANSS scores below 10, and three have scores between 10 and 14, suggesting that five patients could be considered in remission. Our current study primarily examines the influence of two feature sets—acoustic and textual—on classification outcomes, as detailed in Table 4. Although patient interactions may vary, we believe that analyzing these feature sets within the same group of patients is a valid approach. Furthermore, in an additional analysis, we observed a significant association, beyond mere chance, between these feature sets and the PANSS-total score. The analysis involved clustering the 23 patients into 2, 3, and 8 clusters, performed independently. We conducted the experiments twice, representing patients once with textual features and once with acoustic features. The *k*-means algorithm was employed for clustering, and the mutual information (MI) metric was used to assess the relationship between the cluster assignment for each patient and their PANSS-total score. Given that the centroids in the *k*-means algorithm are randomly initialized, we ran each analysis 50 times to ensure reliability. This approach allowed us to report the average MI and its standard deviation. The MI scores, detailed in Table 6, indicate that knowing a patient’s cluster assignment enhances the predictability of their PANSS-total score. These results suggest that the selected features vary in alignment with PANSS scores, reinforcing our primary conclusion that both acoustic and textual features are correlated with symptoms of schizophrenia.

**Table 6:** Mutual information—mean and (standard deviation)—between the variable assigning a cluster for each patient (using *k*-means) and the PANSS-total score.

Feature set	Two clusters	Three clusters	Eight clusters
Textual	0.40 (0.02)	0.78 (0.09)	1.56 (0.06)
Acoustic	0.36 (0.00)	0.65 (0.10)	1.53 (0.07)

**Fig. 1:**
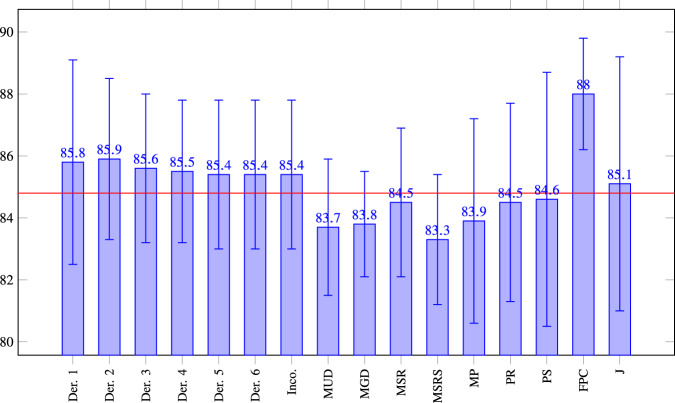
Ablation study: F1 (*y*-axis) scores of the Combined XGBoost classifier by removing one feature from the data at a time, as indicated by the *x*-axis. The red line at 84.8 indicates the F1 value for XGBoost with all features included. The F1 scores the average of five executions, each using a different seed. Der. = Derailment; Inco. = Incoherence.

We performed an ablation study to measure the effect of each feature individually. The results are summarized in Fig. 1. As can be seen, MSRS, MUD and MGD are the most effective features; both are related to the pace of speech. It is noteworthy that removing certain features, primarily textual ones, slightly improves the performance of the classifier. The most significant one is FPC, which measures the level at which the speaker sustains constant pitch. Our hypothesis is that this is mainly a result of overlap in our feature descriptions.

**Fig. 2:**
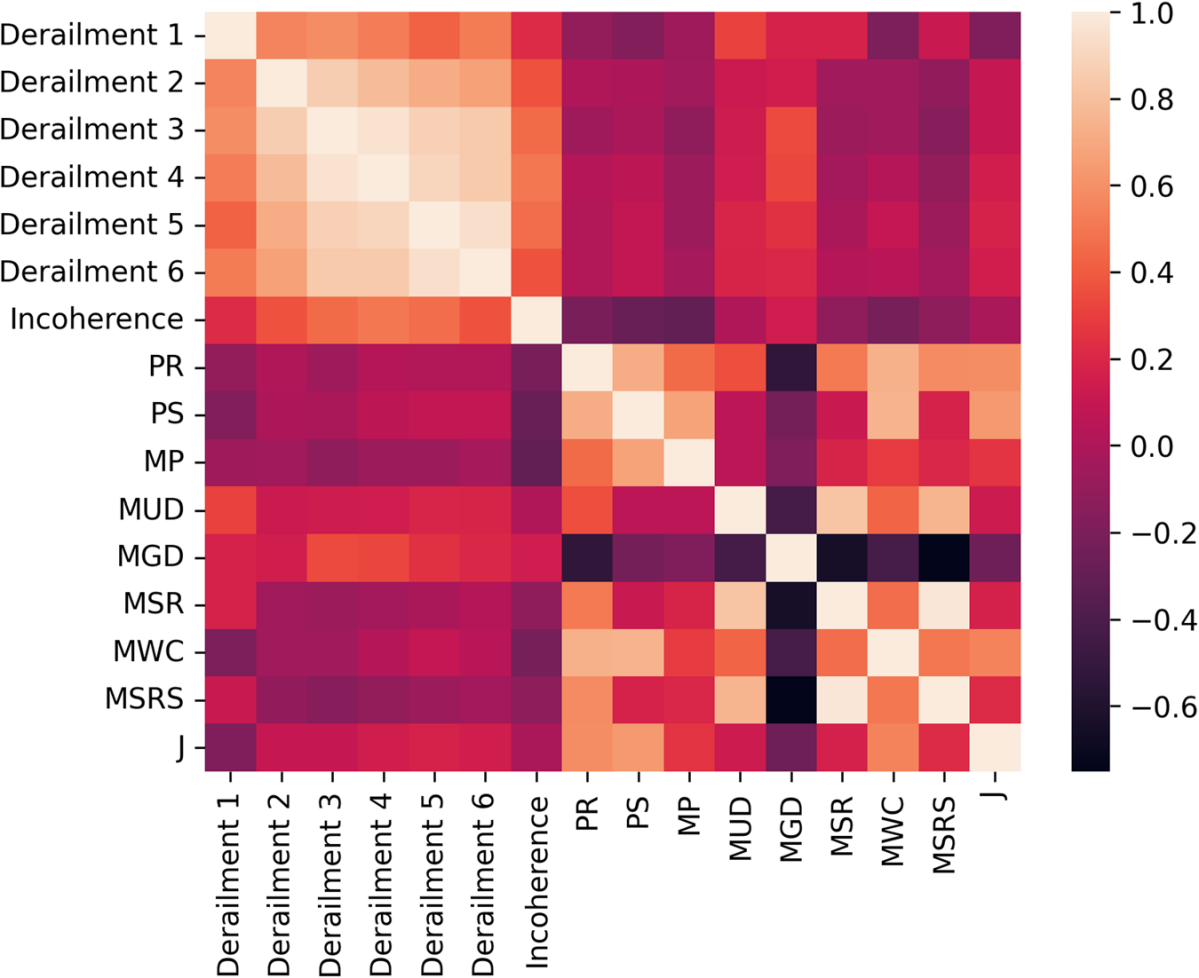
Pearson *p* between all individual features, shown as a heat map.

To measure the correlation between all the individual features, we calculate Pearson *p* for all feature pairs and summarize them in a heat map, as shown in Fig. 2. Unsurprisingly, we see a strong correlation between all the textual derail- ment features, which makes them somewhat redundant for classification. Among the acoustic features, we see a stronger correlation between the standard deviation of the pitch (SP) and the frame pitch correlation (FPC). Generally speaking, both represent the dynamics of the pitch in speech frames. Similarly, and unsurprisingly, the mean spoken ratio (MSR) is strongly correlated with mean spoken ratio samples (MSRS); both represent the ratio between the time in which actual speaking is taking place and the overall time of the response. Naturally, gap duration (MGD) has a negative correlation with all the features that measure speaking duration. However, we do not find any significant correlation between the acoustic features and the textual ones. And, as seen in Table 4, the textual features did not contribute added information for classification not already covered by the acoustic prosodic features.

## Conclusion

We have extracted features from two modalities of Hebrew speech produced by schizophrenia patients during interviews and compared it with those of controls. Specifically, we extracted acoustic, prosodic features from the audio signal, as well as textual features of transcriptions of the interview that measure derailment and incoherence. Our main goal was to measure the contribution of each modality to classification performance, when used in combination. Generally speaking, we find that a traditional classification algorithm can nicely separate between the two groups, schizophrenia patients and healthy controls, with best accuracy of about 90%, which is better than the results that have been previously reported. The results also show that the textual features do not add much to classification performance when they are combined with the acoustic features that measure aspects of prosody.

### Supplementary information

Data1

## Data Availability

We have included a dataset as supplementary material, which details the values of all features for each participant, categorized by question.

## References

[1] Aloia MS (1998). Cognitive substrates of thought disorder, II: specifying a candidate cognitive mechanism. Am. J. Psychiatry.

[2] American Psychiatric Association DSM-5 Task Force. *Diagnostic and Sta- tistical Manual of Mental Disorders: DSM-5*. Vol. 5. (American Psychiatric Publishing, Washington, DC, 2013).

[3] Andreasen NC (1979). Thought, language, and communication disorders: II. Diagnostic significance. Arch. Gen. Psychiatry.

[4] Cherry, C. in *Disorders of Language: Ciba Foundation Symposium* (eds. A. V. S. de Reuck and M. O’Connor. London: J. & A. Churchill), p. 294 (Archives of Internal Medicine, American Medical Association, 1964).

[5] Spoerri TH (1966). Speaking voice of the schizophrenic patient. Arch. Gen. Psychiatry.

[6] Cohen AS, Kim Y, Najolia GM (2013). Psychiatric symptom versus neurocognitive correlates of diminished expressivity in schizophrenia and mood disorders. Schizophr. Res..

[7] Bar, K. et al. “Semantic characteristics of schizophrenic speech”. in *Proceedings of the Sixth Workshop on Computational Linguistics and Clinical Psychology*. June, pp. 84–93. 10.18653/v1/W19-3010 (Association for Computational Linguistics, Minneapolis, MN, 2019).

[8] Kliper, R., Portuguese, S., & Weinshall, D. “Prosodic analysis of speech and the underlying mental state”. in *International Symposium on Pervasive Computing Paradigms for Mental Health*. pp. 52–62 (Springer, 2015).

[9] Kliper, R., Vaizman, Y., Weinshall, D., & Portuguese, S. “Evidence for depression and schizophrenia in speech prosody”. in *Pro- ceedings of the Third ISCA Workshop on Experimental Linguistics*. Athens, Greece, pp. 35–38. https://www.isca-speech.org/archive_v0/exling_2010/papers/el10_085.pdf (2010).

[10] Huang Y-J (2022). Assessing schizophrenia patients through linguistic and acoustic features using deep learning techniques. IEEE Transact Neural Syst. Rehabil. Eng..

[11] Covington MA (2005). Schizophrenia and the structure of language: the linguist’s view. Schizophr. Res..

[12] Hoekert M, Kahn RS, Pĳnenborg M, Aleman A (2007). Impaired recognition and expression of emotional prosody in schizophrenia: review and meta-analysis. Schizophr. Res..

[13] Martínez-Sánchez F (2015). Can the acoustic analysis of expressive prosody discriminate schizophrenia?. Span. J. Psychol..

[14] Alpert M, Rosenberg SD, Pouget ER, Shaw RJ (2000). Prosody and lexical accuracy in flat affect schizophrenia. Psychiatry Res..

[15] Low DM, Bentley KH, Ghosh SS (2020). Automated as- sessment of psychiatric disorders using speech: a systematic review. Laryngoscope Investig. Otolaryngol..

[16] Dickey CC (2012). Prosodic ab- normalities in schizotypal personality disorder. Schizophr. Res..

[17] He F, He L, Zhang J, Li YY, Xiong X (2021). Automatic detection of affective flattening in schizophrenia: acoustic correlates to sound waves and auditory perception. IEEE/ACM Transact. on Au- dio, Speech Lang. Process..

[18] Agurto, C. et al. “Analyzing acoustic and prosodic fluctuations in free speech to predict psychosis onset in high-risk youths”. in *42nd Annual International Conference of the IEEE Engineering in Medicine & Biology Society (EMBC)*. pp. 5575–5579 (IEEE, 2020). 10.1109/EMBC44109.2020.9176841 33019241

[19] Lucarini V (2020). Speech prosody as a bridge between psychopathology and linguistics: the case of the schizophrenia spectrum. Front. Psychiatry.

[20] Lucarini V (2022). Conversational metrics, psychopathological dimensions and self-disturbances in patients with schizophrenia. Eur. Arch. Psychiatry Clin. Neurosci..

[21] Elvevåg B, Foltz PW, Weinberger DR, Goldberg TE (2007). Quantifying incoherence in speech: an automated methodology and novel application to schizophrenia. Schizophr. Res..

[22] Deerwester S, Dumais ST, Furnas GW, Landauer TK, Harshman R (1990). Indexing by latent semantic analysis. J. Am. Soc. Inform. Sci..

[23] Bedi, G. et al. “Automated analysis of free speech predicts psychosis onset in high-risk youths”. *npj Schizophr.* 1, 15030. 10.1038/npjschz.2015.30 (2015). 10.1038/npjschz.2015.30 PMC4849456 27336038

[24] Iter, D., Yoon, J., & Jurafsky, D. “Automatic detection of incoherent speech for diagnosing schizophrenia”. in *Proc. Fifth Workshop on Computational Linguistics and Clinical Psychology: From Keyboard to Clinic*. pp. 136–146 (Association for Computational Linguistics, 2018).

[25] Bojanowski, P., Grave, E., Joulin, A., & Mikolov, T. “Enriching word vectors with subword information”. *Trans. Assoc. Comput. Linguist.* **5**, 135–146 (2017).

[26] Ziv I (2022). Morphological characteristics of spoken language in schizophrenia patients – An exploratory study. Scand. J. Psychol..

[27] Corona-Hernández H, de Boer JN, Brederoo SG, Voppel AE, Sommer IEC (2023). Assessing coherence through linguistic connectives: analysis of speech in patients with schizophrenia-spectrum disorders. Schizophr. Res..

[28] Corcoran CM (2020). Language as a biomarker for psychosis: a natural language processing approach. Schizophr. Res..

[29] Beck AT, Steer RA, Ball R, Ranieri WF (1996). Comparison of beck depression inventories -IA and -II in psychiatric outpatients. J. Personal. Assess..

[30] Gallagher D, Nies G, Thompson LW (1982). Reliability of the beck depression Inventory with older adults. J. Consult. Clin. Psychol..

[31] Hasenson-Atzmon K (2016). Cultural impact on SAD: Social anxiety disorder among Ethiopian and Former Soviet Union immigrants to Israel, in comparison to native-born Israelis. Israel J. Psychiatry.

[32] Weathers, F. W. et al. *The PTSD checklist for DSM-5 (PCL- 5)*. Scale available from the National Center for PTSD at www.ptsd.va.gov. (2013).

[33] Blevins CA, Weathers FW, Davis MT, Witte TK, Domino JL (2015). The posttraumatic stress disorder checklist for *DSM-5* (PCL-5): development and initial psychometric evaluation. J. Trauma. Stress.

[34] Bensimon M (2013). Elaboration on posttraumatic stress disorder diagnostic criteria: a factor analytic study of PTSD exposure to war or terror. Israel J. Psychiatry.

[35] Spielberger, C. D., Gorsuch, R. L. & Lushene, R. E. *STAI Manual for the State-Trait Anxiety Inventory (“self-evaluation questionnaire”)* (Palo Alto: Consulting Psychologists Press,1970).

[36] Knight RG, Waal-Manning HJ, Spears GF (1983). Some norms and reliability data for the State-Trait Anxiety Inventory and the Zung Self-Rating Depression scale. Br. J. Clin. Psychol..

[37] Barnes LLB, Harp D, Jung WS (2002). Reliability generalization of scores on the Spielberger state-trait anxiety inventory. Educ. Psychol. Measure..

[38] Saka N, Gati I (2007). Emotional and personality-related aspects of persistent career decision-making difficulties. Journal of Vocational Behavior.

[39] Østergaard SD, Lemming OM, Mors O, Correll CU, Bech P (2016). PANSS-6: A brief rating scale for the measurement of severity in schizophrenia. Acta Psychiatr. Scand..

[40] Lin C-H (2018). Early improvement in PANSS-30, PANSS- 8, and PANSS-6 scores predicts ultimate response and remission during acute treatment of schizophrenia. Acta Psychiatr. Scand..

[41] Katz G (2012). A comparative study of Arab and Jewish patients admitted for psychiatric hospitalization in Jerusalem: The demographic, psychopathologic aspects, and the drug abuse comorbidity. Compr. Psychiatry.

[42] Boersma, P. *Praat: doing phonetics by computer*. Computer program. http://www.praat.org (2011).

[43] Grave, E., Bojanowski P., Gupta P., Joulin A., & Mikolov T. “Learning word vectors for 157 languages”. in *Proc. International Conference on Language Resources and Evaluation (LREC 2018)*. pp. 3483–3487 (European Language Resources Association (ELRA), 2018).

[44] Adler, M. “Hebrew morphological disambiguation: an unsupervised stochastic word-based approach”. PhD thesis. Beer-Sheva, Israel: Ben-Gurion University of the Negev (2007).

[45] More, A. & Tsarfaty, R. “Data-driven morphological analysis and disambiguation for morphologically rich languages and universal dependencies”. in *Proc. 26th International Conference on Com- putational Linguistics (COLING)*. Osaka, Japan, pp. 337–348. https://aclanthology.org/C16-1033.pdf (2016).

[46] Chen, T. & Guestrin, C. “XGBoost: A scalable tree boosting system”. in *Proc. 22nd ACM SIGKDD International Conference on Knowledge Discovery and Data Mining*. pp. 785–794 (Association for Computing Machinery, 2016).

[47] Liaw A (2002). Classification and regression by ran- domForest. R News.

[48] Cortes C, Vapnik V (1995). Support-vector networks. Machine Learn..

[49] Cangemi, F. et al. “Content-free speech activity records: interviews with people with schizophrenia”. in *Language Resources and Evaluation*, pp. 1–25 (Springer, 2023).

